# Methylprednisolone therapy induces differential metabolic trajectories in severe COVID-19 patients

**DOI:** 10.1128/msystems.00726-23

**Published:** 2023-10-24

**Authors:** Victor I. Mwangi, Rebeca L. A. Netto, Mayla G. S. Borba, Gabriel F. Santos, Gesiane S. Lima, Lucas S. Machado, Michael N. Yakubu, Fernando F. A. Val, Vanderson S. Sampaio, Marco A. Sartim, Hector H. F. Koolen, Allyson G. Costa, Maria C. M. Toméi, Tiago P. Guimarães, Andrea R. Chaves, Boniek G. Vaz, Marcus V. G. Lacerda, Wuelton M. Monteiro, Luiz G. Gardinassi, Gisely C. Melo

**Affiliations:** 1 Programa de Pós-Graduação em Medicina Tropical, Universidade do Estado do Amazonas (UEA), Manaus, Amazonas, Brazil; 2 Fundação de Medicina Tropical Heitor Vieira Dourado (FMT-HVD), Manaus, Amazonas, Brazil; 3 Laboratório de Cromatografia e Espectrometria de Massas, Instituto de Química, Universidade Federal de Goiás, Goiânia, Goiás, Brazil; 4 Programa de Pós-Graduação em Ciência da Saúde, Universidade Federal do Amazonas (UFAM), Manaus, Amazonas, Brazil; 5 Programa de Pós-Graduação em Ciências do Movimento Humano, Universidade Federal do Amazonas (UFAM), Manaus, Amazonas, Brazil; 6 Instituto Todos pela Saúde, São Paulo, São Paulo, Brazil; 7 Pró-reitoria de Pesquisa e Pós-graduação, Universidade Nilton Lins, Manaus, Amazonas, Brazil; 8 Grupo de Pesquisa em Metabolômica e Espectrometria de Massas, Universidade do Estado do Amazonas, Manaus, Amazonas, Brazil; 9 Programa de Pós-Graduação em Imunologia Básica e Aplicada, Instituto de Ciências Biológicas, Universidade Federal do Amazonas (UFAM), Manaus, Amazonas, Brazil; 10 Diretoria de Ensino e Pesquisa, Fundação Hospitalar de Hematologia e Hemoterapia do Amazonas (HEMOAM), Manaus, Amazonas, Brazil; 11 Escola de Enfermagem de Manaus, Universidade Federal do Amazonas (UFAM), Manaus, Amazonas, Brazil; 12 Programa de Pós-graduação em Ciências Aplicadas à Hematologia (PPGH-UEA/HEMOAM), Manaus, Amazonas, Brazil; 13 Instituto de Patologia Tropical e Saúde Pública, Universidade Federal de Goiás (UFG), Goiânia, Goiás, Brazil; 14 Instituto Leônidas & Maria Deane/Fundação Oswaldo Cruz (ILMD/Fiocruz Amazônia), Manaus, Amazonas, Brazil; Third Institute of Oceanography Ministry of Natural Resources, Xiamen, China

**Keywords:** SARS-CoV-2 virus, COVID-19, profile, metabolomics, methylprednisolone, corticosteroid

## Abstract

Corticosteroids have become a choice for managing severe COVID-19, but the molecular mechanisms behind the response after corticosteroid administration remain incompletely understood. In order to unravel this, comparisons between temporal metabolic profiles in the plasma samples of methylprednisolone (MP)- and placebo-treated COVID-19 patients were performed at different time points. The patient plasma samples used were obtained from a double-blind, randomized, placebo-controlled Phase IIb clinical trial performed on severe COVID-19 patients in the Brazilian Amazon, where the patients received placebo or 0.5 mg/kg MP intravenously twice daily for 5 days. The MP treatment reduced the number of metabolites in the plasma of patients during follow-up. The longitudinal changes in the MP group were in eight metabolic pathways related to steroid hormones and eicosanoids. Direct comparison between the two groups, revealed differences at baseline, which peaked 5 days after initiation of MP treatment. The metabolic pathways differing between the two groups over time included galactose metabolism, glucose and gluconeogenesis, N-glycan metabolism, and prostaglandin formation from arachidonate. Deoxy-galactose, prostaglandin H2, sphingosine, and sphinganine exhibited differential trajectories by day 14 after initiating the MP treatment. Survival of MP-treated COVID-19 patients was associated with modulation of tryptophan metabolism and diminished levels of oxidized glutathione. Network analysis revealed that MP treatment is highly associated with alterations in pathways reflecting eicosanoid metabolism, such as arachidonic acid and prostaglandins. Curiously, there is a crosstalk between metabolomics, biochemistry, and cytokine components. Treatment of systemic and inflammatory conditions induced by SARS-CoV-2 viral infections with methylprednisolone modulates metabolic activity associated with tryptophan and inflammatory lipids.

**IMPORTANCE:**

The SARS-CoV-2 virus infection in humans induces significant inflammatory and systemic reactions and complications of which corticosteroids like methylprednisolone have been recommended as treatment. Our understanding of the metabolic and metabolomic pathway dysregulations while using intravenous corticosteroids in COVID-19 is limited. This study will help enlighten the metabolic and metabolomic pathway dysregulations underlying high daily doses of intravenous methylprednisolone in COVID-19 patients compared to those receiving placebo. The information on key metabolites and pathways identified in this study together with the crosstalk with the inflammation and biochemistry components may be used, in the future, to leverage the use of methylprednisolone in any future pandemics from the coronavirus family.

## INTRODUCTION

Early detection and effective treatment of severely ill patients with coronavirus disease 2019 (COVID-19) has been a major challenge. As a result, coordinated initiatives in drug repositioning, and the development of new drugs and vaccines, have been carried out in the management of this disease. Considering the inflammatory and systemic nature of COVID-19, corticosteroids have become a choice for managing severe COVID-19 (1–3). However, the use of corticosteroids against COVID-19 has been controversial (4, 5). Some randomized clinical trials have demonstrated the benefits of corticosteroid therapy for patients with COVID-19, with better prognosis and clinical results (2, 6, 7). Anti-inflammatory therapy given too early in the course of the disease could be harmful, whereas given at the right time, it would reduce inflammation, improving oxygenation and survival (8).

In recent years, many technological strategies emerged to provide insights into an organism’s metabolic response to a biological perturbation such as an infection. Untargeted metabolomics via liquid chromatography-tandem high-resolution mass spectrometry (LC-HRMS) has promoted significant advances in the study of the molecular profile of different diseases (9). The diverse human metabolome may be altered by endogenous and external influences, ultimately leading to dysregulations that have been associated with a variety of diseases and medications (10). Metabolomics has also facilitated the identification of potentially useful prognostic and diagnostic biomarkers of disease (11).

COVID-19 infection and the cascades associated with the viral infection cause shifts in many metabolic pathways associated with age, sex, disease severity, and fatal outcomes (10, 12–14). Metabolic profiling has been improving the understanding of the disease, the effect of drugs and vaccination, patient recovery, long-term sequelae, and the impact on gut microbiome during COVID-19 (11, 15, 16).

It is of immediate clinical importance that the benefits or risks of corticosteroids in managing COVID-19 be evaluated and understood. In the present study, we performed an untargeted metabolomic analysis of severe COVID-19 patients participating in a randomized controlled trial designed to test the efficacy of an intravenous methylprednisolone (MP) treatment regimen in reducing mortality. Here, we discuss the findings of longitudinal analysis of plasma metabolites along the course of the trial, in addition to integrating metabolomics with other data types, including blood cell counts, markers of tissue damage, and cytokines.

## MATERIALS AND METHODS

### Study participants and sample processing

The COVID-19-infected participants in this study were drawn from the MetCOVID study (1), a parallel, double-blind, randomized, placebo-controlled Phase IIb clinical trial, that assessed the efficacy of MP in treating hospitalized patients with suspected SARS-CoV-2 infection. Individuals with COVID-19 were admitted at the *Hospital e Pronto-Socorro Delphina Rinaldi Abdel Aziz*, in Manaus, Western Brazilian Amazon. The hospital was the largest public reference unit for the treatment of severe COVID-19 cases in the city. The participants were administered with either intravenous sodium succinate MP (0.5 mg/kg) or placebo (saline solution) twice daily for 5 days. As per hospital protocol, all patients meeting acute respiratory distress syndrome criteria also preemptively received intravenous ceftriaxone (1 g twice daily for 7 days) plus azithromycin (500 mg once a day for 5 days) or clarithromycin (500 mg twice daily for 7 days), starting on day 1. The participant selection criteria and treatment regime have been described elsewhere (1).

At inclusion, adult patients were excluded if they had a history of hypersensitivity to MP, were living with human immunodeficiency virus or AIDS, had a history of chronic use of corticosteroids or immunosuppressive agents, were pregnant or breastfeeding, or had decompensated cirrhosis or chronic renal failure. After confirmation of SARS-CoV-2 infection by RT-qPCR testing of nasopharyngeal swabs, blood was collected from patients and the separated plasma was stored at −80°C. The plasma samples were collected at the day of inclusion (D1) before treatment, day 5 (D5), day 7 (D7), day 11 (D11), and day 14 (D14) after the start of treatment for metabolomic analysis.

For this study, we selected samples from the MetCOVID participants (from both MP and placebo arms) that tested positive for SARS-CoV-2 infection by RT-qPCR test, had no previous use of corticosteroids, and had completed their 5 days MP or placebo treatment.

### Cytokine quantification

Serum levels of soluble immunological mediators (IL-1β, IL-2, IL-4, IL-6, IL-8, IL-10, IL-12p70, IL-17A, TNF, and IFN-γ) were measured in samples using a BD cytometric bead array (CBA) Human Inflammatory Cytokine and Human Cytokine Th1/Th2/Th17 BD kits (BD Biosciences, San Diego, CA, USA), according to the manufacturer’s instructions. The cytokine beads were counted using a flow cytometer (FACSCanto II; BD Biosciences), and analyses were performed using FCAP Array (3.0) software (BD Biosciences). The determined cytokine concentrations (pg/mL) were used in the subsequent analysis and data integration.

### Untargeted metabolomics

Untargeted metabolomics data were performed as described (17). Metabolites were extracted from plasma samples using acetonitrile (2:1, vol/vol). Stable isotopes caffeine-¹³C3, tyrosine-^15^N, and progesterone-d^9^ were used as internal standards. LC-MS/MS analysis was performed with an HPLC-UV, 1220 Infinity (Agilent Technologies, Santa Clara, CA, USA) coupled with Q Exactive hybrid Quadrupole-Orbitrap high-resolution mass spectrometer (Thermo Fisher, Waltham, MA, USA). Reverse phase C18 chromatography was performed with Zorbax Eclipse Plus C18 column (4.6 × 150 mm^2^, 3.5 µm Agilent) and positive electrospray ionization.

### Untargeted metabolomics (gradient elution and mass spectrometer settings)

All samples were analyzed using a gradient elution program. Pooled samples were used as quality control and included in every batch of samples. The binary mobile phases were water-0.5% formic acid with 5 mM ammonium formate (A), and acetonitrile (B). Their gradient elution started with 20% (B) for 5 min, then linearly increased to 100% (B) in 30 min and kept constant for 8 min in 100% (B). The eluent was restored to the initial conditions in 4 min to re-equilibrate the column and held for the remaining 8 min. The flow rate was kept at 0.5 mL min^−1^. The injection volume for analysis was 3 µL, and the column temperature was set at 35°C. The electrospray ionization operated at the following settings: spray voltage 3.5 kV; capillary temperature: 269°C; S-lens RF level 50 V; sheath gas flow rate at 53 L min^−1^; auxiliary gas flow rate at 14 L min^−1^; and sweep gas flow rate 3 L min^−1^. The high-resolution mass spectrometry was obtained under full MS/dd-MS2 mode. The mass range in the full MS scanning experiments was *m/z* 80 to 1,200. The max IT was set at 200 ms, and AGC target was set at 1 × 10^6^. For fragmentation acquisition, the top 5 (TopN, 5, loop count 5) most abundant precursors were sequentially transferred into the C-Trap (AGC target 1 × 10^5^; max IT 50 ms) for collision. The collision energy for target analytes was 20, 30, and 35 eV. Resolving power was set at 140,000 and 70,000 for full MS and dd-MS2 acquisitions, respectively.

### Bioinformatics and statistical analyses

*Asari* software was used to process raw data and extract metabolite features (18). Data were log_2_ transformed and features were filtered out by 50% presence in all samples. The *mummichog* software was used to predict the activity of metabolic pathways and networks (19). Fragment similarity searches were performed with METLIN Gen2 (https://metlincloud2.massconsortium.com/) (20) and MyCompoundID (http://www.mycompoundid.org/) (21).

Differential abundance was evaluated with moderated t test or moderated F test (ANOVA) using the *limma* R package. Additional statistics included Tukey or Kruskal-Wallis multiple-comparison test. False discovery rate (FDR) was calculated with the Benjamini and Hochberg method. For the network analysis, all data from D1 were compiled and metabolomics data were reduced to 16 clusters using correlation metrics and accounting for retention time as described previously (22). Subsequently, cluster activities were used in Spearman correlation analysis with individual blood cell counts, and quantification of cytokines and biomarkers of inflammation and tissue damage. Significant associations (*P* < 0.05) were retained for visualization using Cytoscape v3.10. We used gene set enrichment analysis to compare metabolic clusters between MP versus placebo.

## RESULTS

### Demographic characteristics of individuals with COVID-19

Fifty-two controls (placebo group) and 49 MP-treated patients, totaling 101, met our selection criteria. The two groups at baseline had no significant difference between demographics, clinical, and laboratory parameters. The duration of illness until inclusion was at a median of 11.5 days (Table 1). Overall, the proportion of males was higher than females (68.3% vs 31.7%), in both groups. The age distribution was similar between placebo and MP-treated groups, with a median age of 60 years. There was also no significant difference in the ethnic distribution of the patients, with most in both groups being of self-declared admixed racial heritage (Table 1).

**TABLE 1:** Baseline demographic, clinical, and laboratory results at randomization^*a*^

Characteristics	Total (*n* = 101)	MP (*n* = 49)	Placebo (*n* = 52)	*P* value
Age, years, median (IQR)	60 (49–69)	60 (46–69)	60 (50.5–72)	0.646
BMI, kg/m^2^, median (IQR)	28 (25.4–31.0)	27.8 (24.6–31.1)	28.4 (25.8–31.0)	0.409
Sex				
Female, *n*/*N* (%)	32 (31.7)	15 (30.6)	17 (32.7)	0.822
Male, *n*/*N* (%)	69 (68.3)	34 (69.4)	35 (67.3)	
Race, *n*/*N* (%)				
White	9 (8.9)	3 (6.1)	6 (11.5)	0.195
Mixed race (Brown)	80 (79.2)	43 (87.8)	37 (71.2)	
Black	6 (5.9)	1 (2.0)	5 (9.6)	
Amerindian/Indigenous	6 (5.9)	2 (4.1)	4 (7.7)	
Days from illness onset to randomization, median (IQR)	11.5 (8–15)	12 (8–15)	11 (8–14)	0.416
Pre-existing morbidities, *n*/*N* (%)	92/101 (91.1)	46/49 (93.9)	46/52 (88.5)	0.340
Hypertension	55/92 (59.8)	26/46 (56.52)	29/46 (63.0)	0.524
Obesity	34/92 (37.0)	16/46 (34.8)	18/46 (39.1)	0.666
Diabetes	27/92 (29.4)	11/46 (23.9)	16/46 (34.8)	0.252
Alcohol use	19/92 (20.7)	12/46 (26.1)	7/46 (15.2)	0.344
Liver disease	10/92 (10.9)	2/46 (4.4)	8/46 (17.4)	0.074
Smoking (currently)	4/92 (4.4)	3/46 (6.5)	1/46 (2.2)	0.753
Coronary heart disease	9/92 (9.8)	4/46 (8.9)	5/46 (10.9)	0.726
Chronic respiratory disease	4/92 (4.4)	2/46 (4.4)	2/46 (4.4)	>0.999
Previous TB	3/92 (3.3)	2/46 (4.4)	1/46 (2.2)	0.502
TB in treatment	1/92 (1.1)	1/46 (2.2)	0/46 (0)	>0.999
Chronic hematological disease	2/92 (2.2)	2/46 (4.4)	0/46 (0)	0.495
Neurological disease	2/92 (2.2)	1/46 (2.2)	1/46 (2.2)	>0.999
Medication use, *n*/*N* (%)	101/101 (100)	49/49 (100)	52/52 (100)	>0.999
Antibiotics	76/101 (75.3)	38/49 (77.6)	38/52 (73.1)	0.603
Azithromycin	57/73 (78.1)	27/37 (73.0)	30/36 (83.3)	0.285
ACE inhibitors	48/101 (47.5)	19/49 (38.8)	29/52 (55.8)	0.087
Bronchodilators	10/101 (9.9)	4/49 (8.2)	6/52 (11.5)	0.570
Statins	5/101 (5.0)	2/49 (4.1)	3/52 (5.8)	>0.999
Calcium blockers	3/101 (3.0)	3/49 (6.1)	0/52 (0)	0.111
Other remedies	99/101 (98.0)	47/49 (95.9)	52/52 (100)	0.141

^*a*^ BMI, body mass index; IQR, interquartile range; MP, methylprednisolone; N, sample size number; TB, tuberculosis.

### Differential metabolic activity in the two treatment groups over time

**Fig 1:**
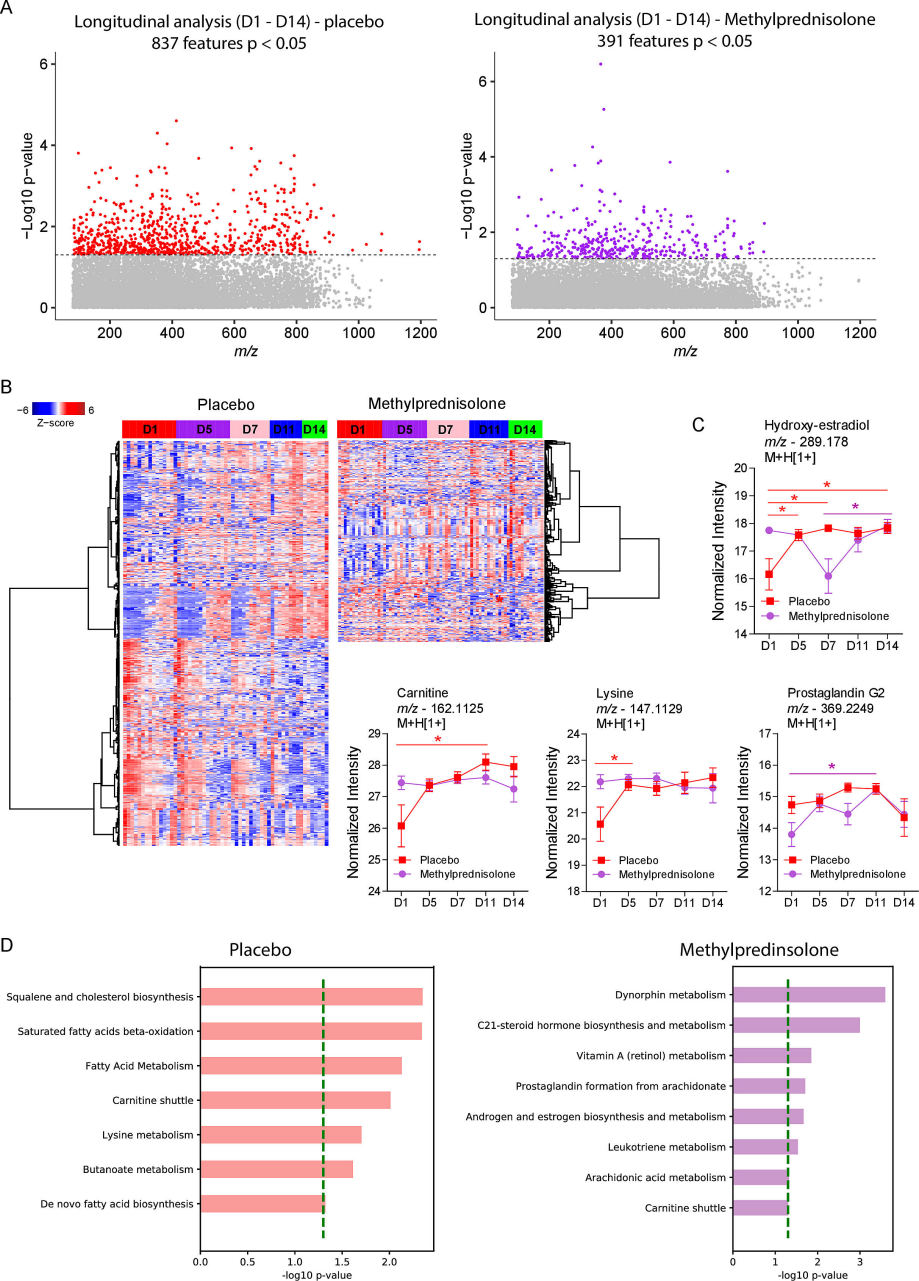
Plasma metabolic signatures in the placebo and MP groups from D1 to D14. (**A**) Manhattan plot showing the distribution of significant metabolite features after a moderated *F*-test of metabolites in the patients’ plasma samples. The dashed line indicates *P* < 0.05. (**B**) Hierarchical clustering of identified significant metabolite features in the two patient groups. (**C**) Differential abundance analysis of significant metabolites common in the two treatment groups. (**D**) Mummichog pathway analysis of significant features per group. A multiple comparison test was used for between-day comparisons. *P < 0.05.

The LC-MS/MS data processing resulted in 11,682 metabolite features measured in the whole data set. Analysis along the course of 14 days after initiating treatment identified 837 and 391 differentially abundant *m/z* features in the placebo and MP groups, respectively (Fig. 1A). Heat maps of significant *m/z* features showed more perturbations in the metabolomes of placebo group, which is formed by two large clusters that followed inverse dynamics along the 14 days of follow-up (Fig. 1B). At the same time, the MP group displayed three main clusters, one whose abundances were mostly reduced at D5, one that increased abundance at D14 and one that reduced abundance at D14 (Fig. 1B). Annotated metabolites include hydroxyestradiol, whose abundance increased at all time points when compared before treatment in the placebo group and differed significantly between D14 and D7 in the MP group (Fig. 1C). Other examples of metabolites differing from the placebo group include carnitine, which increased considerably between D1 and D11, and lysine, which increased considerably by D5 before stabilizing in the placebo group. Prostaglandin G2 levels increased significantly in the MP group between D1 and D11 before falling on D14. Mummichog analysis of the untargeted metabolomics data predicted the activity of diverse metabolic pathways. Placebo treatment was characterized by perturbations in squalene and cholesterol biosynthesis, saturated fatty acid beta-oxidation, fatty acid metabolism, lysine metabolism, and butanoate metabolism, whereas MP treatment was associated with dynorphin metabolism, C21-steroid biosynthesis and metabolism, androgen and estrogen metabolism, arachidonic acid metabolism, prostaglandin formation from arachidonate, and leukotriene metabolism (Fig. 1D). Interestingly, significant enrichment of carnitine shuttle was common to both groups.

### Comparison of metabolites between different groups of treatment over time

**Fig 2:**
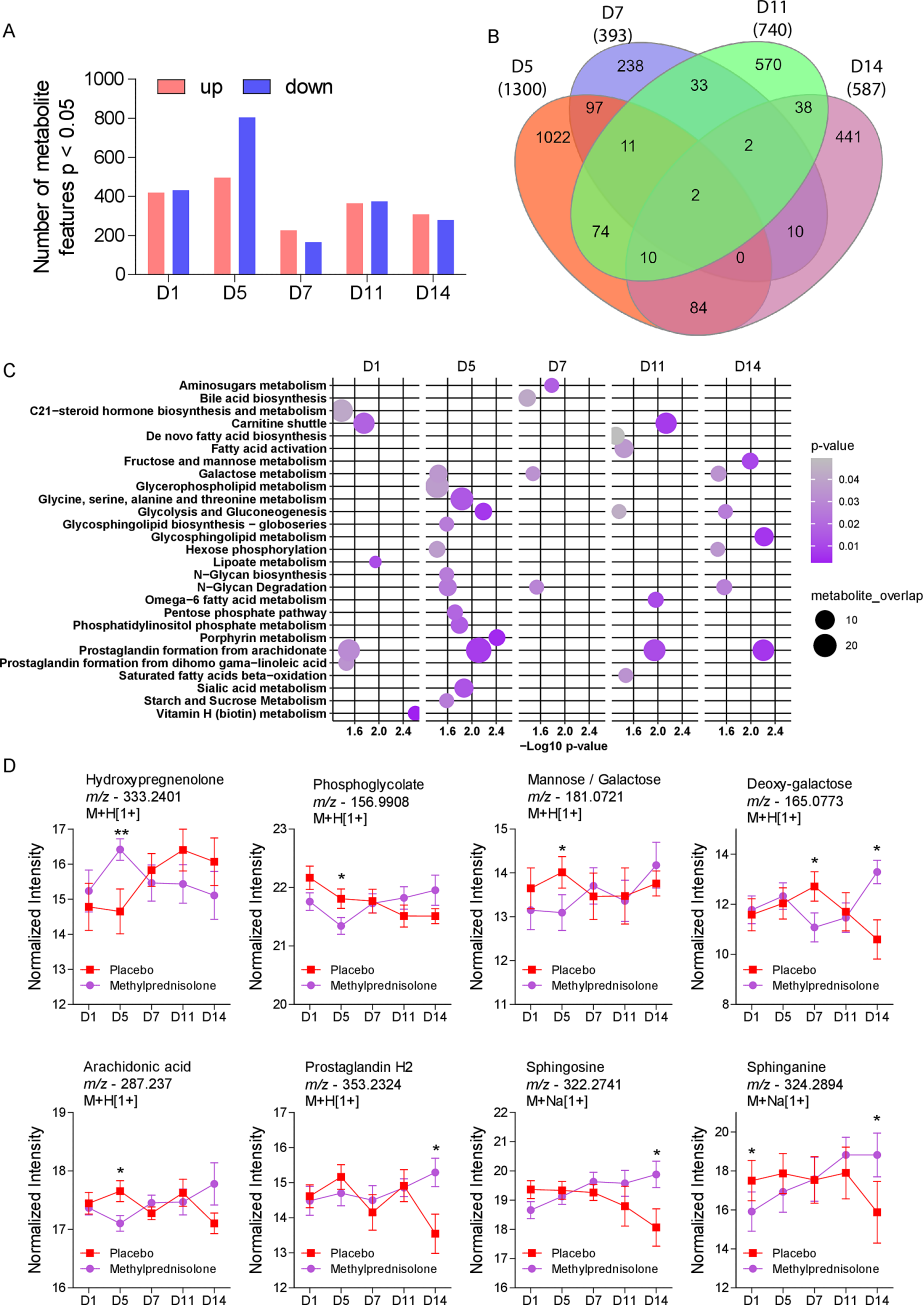
Longitudinal comparison between placebo or MP-treated COVID-19 patients. (**A**) Differential abundance of the significant metabolite features found from D1 to D14. (**B**) Venn diagram of differentially abundant metabolites on D5 to D14. The numerical values are indicative of the number of different metabolites detected at different time points. The color code is illustrative of the time points: D5-Orange, D7-Indigo, D11-Green, and D14-Purple. (**C**) Pathway enrichment analysis of significant metabolite features for each of the sampling time points. (**D**) Temporal analysis of the relative abundance of the significant metabolite features detected in the two patient groups at D1 to D14. *P < 0.05, **P < 0.01.

The overall patient sample size decreased at the respective time points (D1, *n* = 101; D5, *n* = 85; D7, *n* = 62; D11, *n* = 31; and D14, *n* = 21). Although there were no differences in any potential confounding factor such as age, sex, and presence of comorbidities (Table 1), we detected significant differences on the metabolic profiles between the two groups before treatment (Fig. 2A). The most difference was detected at D5, with up to 800 downregulated *m/z* features in the MP group compared to placebo whereas D7 had the lowest number of up- and downregulated *m/z* features (Fig. 2A). To understand whether detected changes at each time point reflect the same *m/z* features, we plotted a Venn diagram that revealed most changes were specific for a determined time point, with D5 and D7 revealing the highest and least number of significant *m/z* features (Fig. 2B), respectively. Pathway enrichment analysis revealed differential activity of several metabolic pathways, and as expected, D5 was associated with a higher number of pathways (Fig. 2C). Moreover, pathways, such as galactose metabolism, glycolysis and gluconeogenesis, and N-glycan degradation, were common in three time points, while prostaglandin formation from arachidonate was commonly enriched for four time points (Fig. 2C). Analysis of metabolites related to these pathways revealed many significant changes at D5, including hydroxypregnolone, phosphoglycolate, mannose/galactose, deoxy-galactose, and arachidonic acid (Fig. 2D). Other differences emerged at D14, including deoxy-galactose, prostaglandin H2, sphingosine, and sphinganine.

### Survival after MP treatment is associated with modulation of tryptophan metabolism

**Fig 3:**
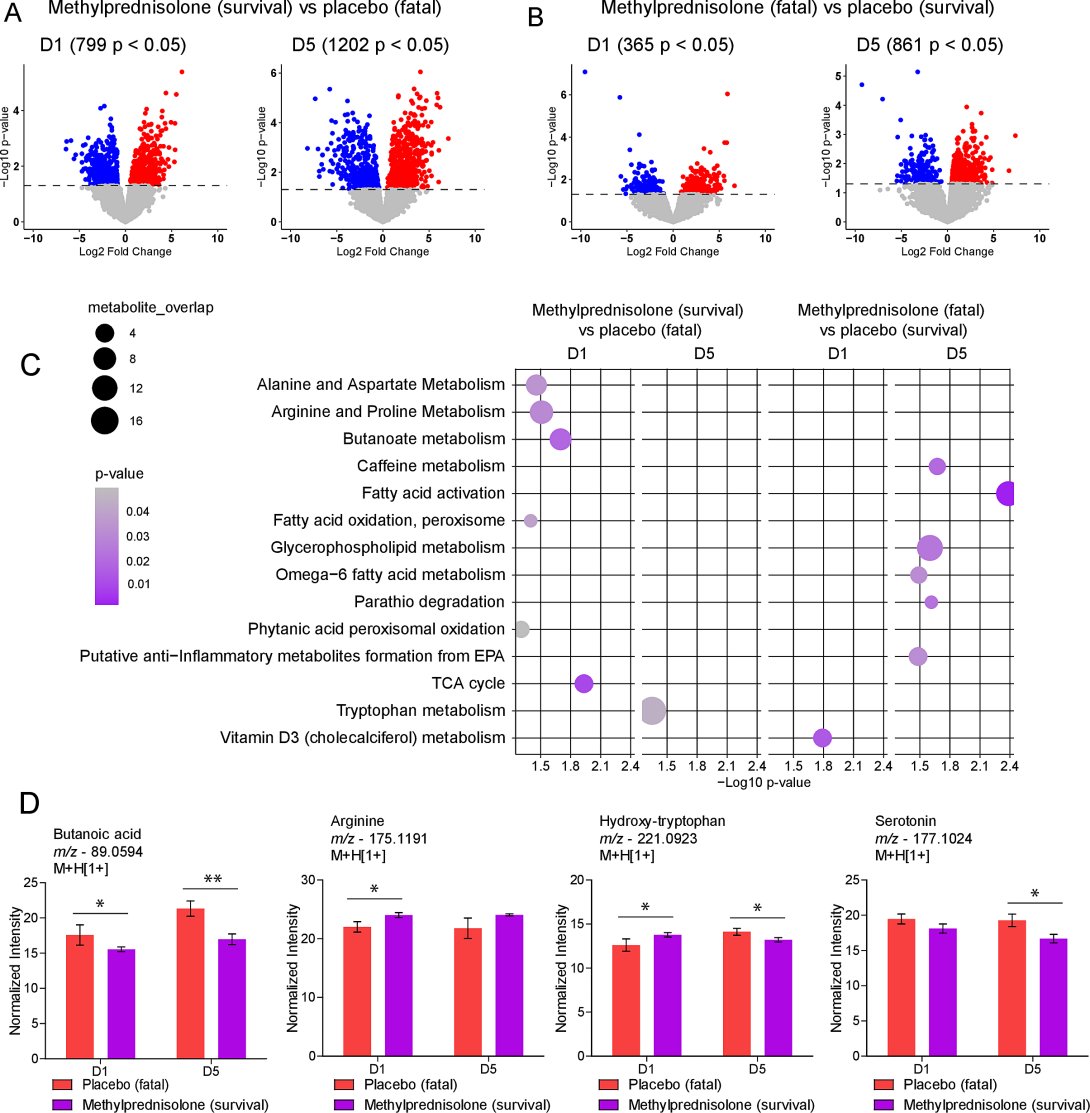
Favorable or detrimental metabolic effects of MP treatment. (**A and B**) Volcano plots comparing two sets of patients at D1 and D5, demonstrating significantly different metabolite features differing of treated survivors and fatalities. The dashed line represents *P* < 0.05. The blue dots represent downregulated, while the red dots represent upregulated features in each comparison. (**C**) Mummichog pathway analysis of significant metabolite features on D1 and D5 for survivors and fatalities in the MP and placebo groups. Pathways that were significant for comparisons of survival and death in the same group of treatment were removed. (**D**) Differential abundance of annotated metabolites identified in treated individuals that survived compared to placebo who died. *P < 0.05, **P < 0.01.

To identify metabolites associated with beneficial or detrimental effects of MP treatment, we compared the metabolic activity between patients that survived up to D14 (placebo = 8; MP = 12), and patients with fatal outcomes before D14 (placebo = 7; MP = 4). Before treatment, patients allocated to MP group that survived COVID-19 displayed 799 differentially abundant *m/z* features, which increased to 1,202 at D5 when compared to patients in the placebo group who died (Fig. 3A). Comparing MP-treated patients who died to placebo-treated patients that survived COVID-19, we found 365 and 861 significant metabolite features at D1 and D5, respectively (Fig. 3B). Pathway enrichment comparison between survival and fatal outcomes before treatment (D1) suggested that patients in our cohorts already differed in pathways such as alanine and aspartate metabolism, arginine and proline metabolism, butanoate metabolism, TCA cycle and phytanic acid oxidation and vitamin D3 metabolism (Fig. 3C). Moreover, survival of patients treated with MP is associated with modulations in tryptophan metabolism, while fatal outcomes of MP treatment are associated with caffeine metabolism, fatty acid activation, glycerophospholipid metabolism, and others as shown in Fig. 3C. Examples of metabolites involved in these pathways include butanoic acid, whose abundance was lower at D1 and D5, as well as arginine, whose abundance at D1 was higher in MP treated patients that survived COVID-19 (Fig. 3D). Of interest, levels of hydroxy-tryptophan (HTP) were higher at D1, but reduced significantly at D5 in MP-treated patients that survived compared to placebo-treated patients who died from COVID-19. In addition, serotonin levels were also reduced at D5 in the surviving MP-treated patients (Fig. 3D).

**Fig 4:**
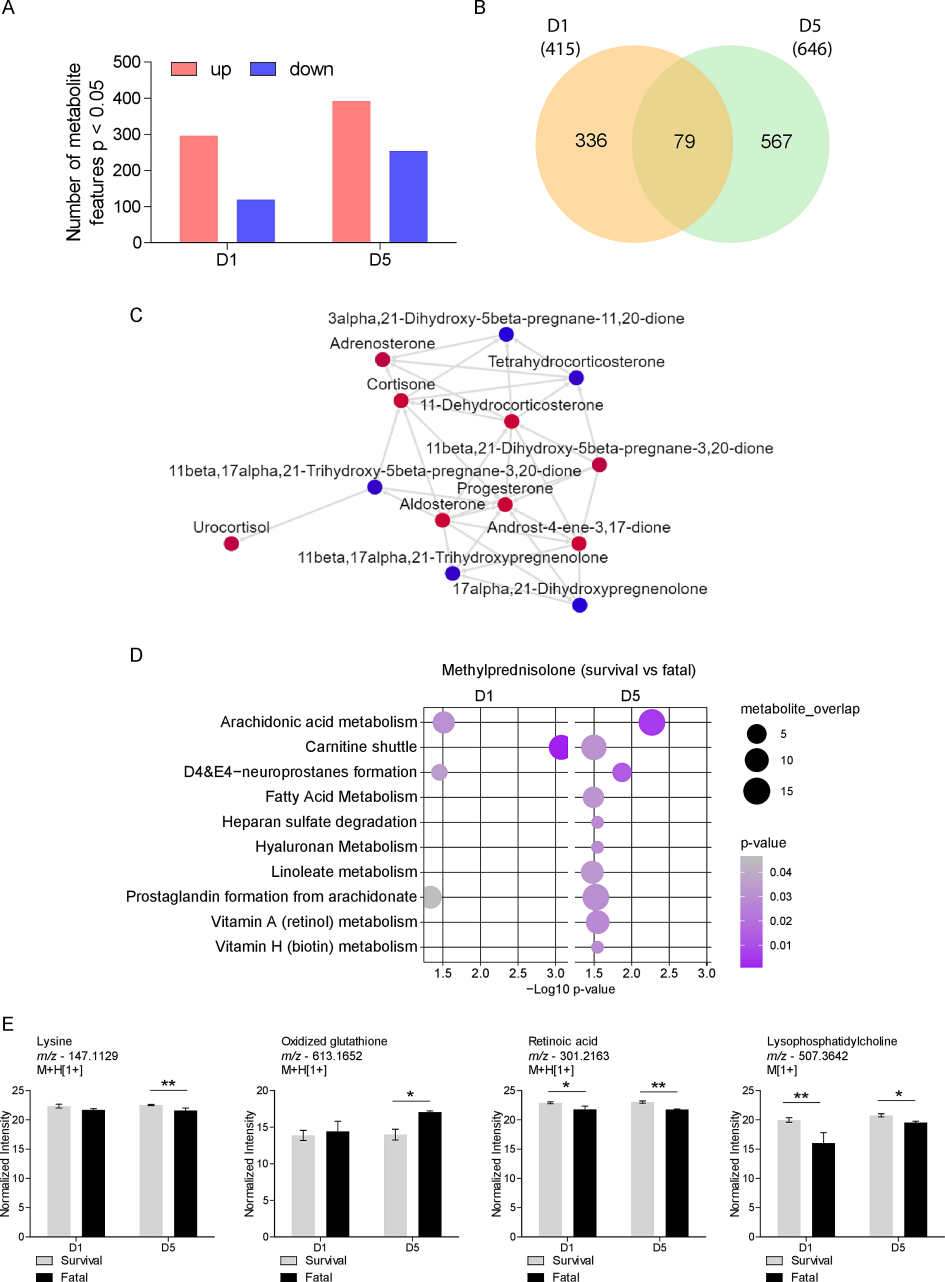
Survival after MP treatment is associated with differences in lipid and vitamin metabolism. (**A**) Differential abundance of metabolite features between MP-treated patients who died before or survived after 14 days at D1 and D5. (**B**) Venn diagram comparing significant metabolite features at D1 and D5. (**C**) Significant metabolic network associated with steroid hormone metabolism enriched by differentially abundant metabolite features at D1. (**D**) Mummichog pathway analysis of significant metabolite features at D1 and D5 for MP-treated patients who died before or survived after 14 days. (**E**) Differential abundance of annotated metabolites identified in MP-treated patients that survived compared to those who died before 14 days of treatment. *P < 0.05, **P < 0.01.

To further explore treatment effects, we compared the metabolomes of patients treated with MP for D1 and D5, focusing on those who died before D14, and those who survived. We observed 296 upregulated and 119 downregulated metabolic features on D1, and 392 upregulated and 254 downregulated features on D5 (Fig. 4A) of which 79 were common between the 2 time points (Fig. 4B). Mummichog analysis predicted the activity of a significant metabolic network at D1 that is related to steroid hormone biosynthesis and metabolism (Fig. 4C). Furthermore, pathway enrichment analysis predicted significant activity of arachidonic acid metabolism, carnitine shuttle, formation of neuroprostanes D4 and E4, and prostaglandin formation from arachidonate in both D1 and D5 (Fig. 4D). Fatty acid metabolism, heparan sulfate degradation, hyaluronan, linoleate, vitamin A, and vitamin H metabolism were enriched only in D5 (Fig. 4D). Annotated metabolites include lysine, whose levels were reduced, and oxidized glutathione whose levels increased on D5 in the MP-treated patients who died (Fig. 4E). The abundance of features annotated as retinoic acid and lysophosphatidylcholine were reduced at both time points in MP-treated patients who died (Fig. 4E).

### Integrated molecular networks associated with MP treatment during COVID-19

**Fig 5:**
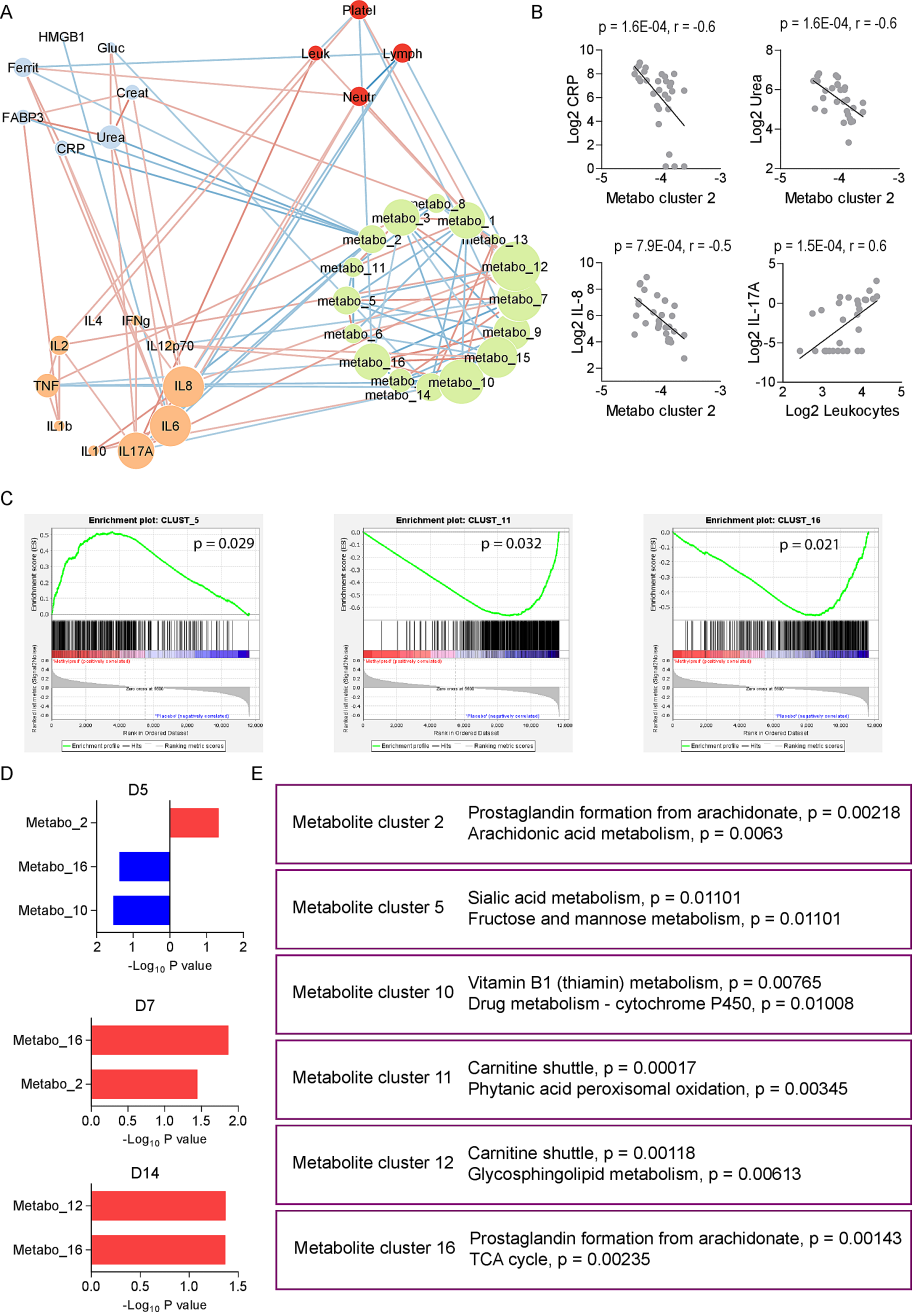
Integrative network analysis of placebo and MP-treated COVID-19 patients. (**A**) Integrative network between laboratory parameters (biochemical and hematological), cytokines, and metabolite clusters on D1. The red lines indicate positive associations and the blue lines represent negative associations. (**B**) Examples of the most significant correlations found in the integrative network. (**C**) Cluster enrichment analysis comparing methylprednisolone versus placebo at D1. (**D**) Cluster enrichment analysis comparing methylprednisolone versus placebo at D5, D7, and D14. (**E**) Pathway enrichment analysis of significant clusters in items (C) and (D).

To understand the molecular effects of MP treatment during COVID-19, we integrated metabolomics data with blood cell counts, cytokines and markers of inflammation, and tissue damage. For that, we first reduced the dimension of the whole metabolomics data into 16 metabolic clusters that were later used to perform Spearman correlation analysis with other data types. Significant associations (*P* < 0.05) resulted in a robust network of 39 nodes linked by 130 edges (Fig. 5A). The most significant associations include negative correlations between metabolite cluster 2 with C-reactive protein, urea, and IL-8, whereas IL-17A was positively correlated with leukocytes (Fig. 5B). We used metabolite clusters to evaluate differences between the groups. Using this strategy, we confirmed that despite very well-matched participants in the trial, we still detected differences between groups before treatment (D1) that are related to sialic acid metabolism (Metabo cluster 5), carnitine shuttle (Metabo cluster 11), and prostaglandin formation (Metabo cluster 16) (Fig. 5C and E). MP treatment was associated with metabolite cluster 2 at D5 (Fig. 5D), which involves prostaglandin formation (Fig. 5E); metabolite clusters 2 and 16 at D7, both of which involve prostaglandin formation, but also arachidonic acid metabolism and TCA cycle; and metabolite clusters 16 and 12 at D14, the former which involves carnitine shuttle and glycosphingolipid metabolism (Fig. 5D and E).

## DISCUSSION

The COVID-19 pandemic presented an unprecedented threat to global public health. Conversely, several therapeutic interventions including corticosteroids have been recommended for use among COVID-19 patients, with beneficial effects (23). During viral infection, pathogenic viruses can promote metabolic changes that favor viral survival, alter cell phenotype and function, and cause long-term inflammation and tissue injury (24, 25).

Metabolomics analysis has improved the understanding of how COVID-19 induces host systemic and cellular metabolic changes, revealed metabolic pathways associated with COVID-19 severity, identified diagnosis and prognosis biomarkers, and complemented the knowledge gap underlying clinical manifestations and pathogenesis of COVID-19 (13, 26–28). However, most of these studies focus on the application of metabolomics and/or lipidomics analysis on different categories of COVID-19 patients and gut health in COVID-19.

It has been discussed that metabolite profiles emanating from COVID-19 play a role in abnormal pulmonary function. Metabolites from gut-related choline-trimethylamine N-oxide (TMAO) and carnitine-TMAO pathways influence pulmonary dysfunction (dyspnea and low oxygen saturation levels) during COVID-19 (29), whereas elevated levels of triacylglycerols, phosphatidylcholines, prostaglandin E2, arginine, and decreased levels of betain and adenosine have been linked to pulmonary dysfunction in carbon monoxide diffusing capacity and total lung capacity (30). Therefore, abnormal pulmonary function involves pathways such as arginine biosynthesis, and metabolism of arginine, proline, taurine, hypotaurine, glycerophospholipid, glycerolipid, and sphingolipid. The MetCOVID study identified changes in lung function between the MP and placebo groups by D120 of follow-up (1). Understanding whether and how the aforementioned metabolic changes are related to these lung function changes may help better understand the pathogenesis of long COVID.

It has not yet been elucidated how MP would affect the metabolome of COVID-19 patients. To the best of our knowledge, this is the first study showing how the use of MP during COVID-19 infection longitudinally influences the metabolite profile, affecting clinical outcomes. The significant metabolites identified in our cohort generally belonged to 15 pathways. These pathways generally represented dysregulation in fatty acid/lipid, amino acid, energetic, and hormonal metabolism. While the MP-treated group had predominantly hormonal, retinol, and lipid metabolism dysregulations, the placebo-treated patients manifested dysregulations predominantly in the lipid and amino acid pathways, corroborating findings reported elsewhere (28, 31). Interestingly, there was significant alteration in the carnitine shuttle in both groups suggesting interference in mitochondrial activity, disease severity, and progression (17, 27, 32).

The observed sharp rise in the number of downregulated metabolites could partially be attributed to the fast onset of action of the intravenous MP dose. Similarly, the sharp decline in metabolites detected on the D7 samples could be due to the drug’s short elimination half-life (about 0.25–1.9 h) (33). From our study, infection by the virus was associated with significant activity in multiple pathways such as vitamin H metabolism, C21-steroid hormone biosynthesis and metabolism, the carnitine shuttle, and prostaglandin formation from polyunsaturated acids (arachidonate and dihomo gamma-linoleic acid) among others. This linked the use of MP with dysregulation in the carnitine and steroid hormone biosynthesis, which agreed with another study reporting modulation of COVID-19 dysregulation by glucocorticoid treatment (34).

The up-scaled activity in the galactose metabolism, glycolysis and gluconeogenesis, starch and sucrose metabolism, and pentose phosphate pathway observed in the D5 analysis are synonymous with the MP effects on insulin-glucagon homeostasis (33). Phosphatidylinositol phosphate metabolism was active, driving endosomal fusion, sorting, motility, autophagy, cytokinesis, regulated exocytosis, and signal transduction through phosphatidylinositol 3-phosphate and its effectors (35). Significant phosphatidylinositol and lysophosphatidylinositol metabolic alterations have previously been described in recovered SARS patients treated with high-dose MP (36). This is consistent with our findings of dysregulation in the phosphatidylinositol phosphate metabolism pathway following MP treatment, implying that a high daily dose of MP can cause systemic effects linked with serum metabolic changes.

In both groups, significant dysregulation was observed in endogenous steroids (hydroxyl-estradiol and hydroxypregnenolone), amino acid derivatives (carnitine), amino acids (lysine), eicosanoids (prostaglandin G2 and prostaglandin H2), glycolic acid derivative and DNA damage products (phosphoglycolate) (37), hexoses (mannose/galactose and deoxy-galactose), and fatty acids (Arachidonic acid). Additionally, amino alcohol lipids (sphingosine and sphinganine) were also dysregulated. The intensity of these metabolites changed longitudinally among the groups. Both corticosteroids and sex hormones are produced from cholesterol through the same steroidogenic pathway (38). This could explain why MP therapy was associated with significant changes in the patients’ steroid levels (hydroxyl-estradiol and hydroxypregnenolone). Interestingly, we also found differences in steroid hormone biosynthesis and metabolism between MP-treated patients who died before or survived after 14 days, suggesting that intrinsic alterations in endogenous steroid hormone metabolism might influence the treatment of COVID-19 patients with MP. It is suggested that corticosterone biosynthesis characterized by increased 21-hydroxypregnenolone, an important intermediary in the production of corticosterone, may be a preventive mechanism against SARS-CoV-2 infection (12). Our findings show that MP treatment affects cellular membrane components such as prostaglandin G2, sphinganine, and sphingosine. Prostaglandins (PG) are a group of physiologically active lipid molecules having a variety of hormone-like properties and function as autocrine or paracrine factors to their target cells at the site of their secretion, where they promote or resolve an inflammation (39). These lipid autacoids are produced from arachidonate by the activity of cyclooxygenase isoenzymes (39), thus explaining the initially low arachidonic acid levels in MP-treated patients. These low arachidonate levels could be attributed to PG biosynthesis. Serum sphingosine-1-phosphate (S1P) levels have been found to be higher in COVID-19 patients (40). This metabolite has been shown to induce physiological processes like immunological responses and endothelial barrier integrity (41). However, treatment with MP did not suppress S1P levels and its bioactive pro-inflammatory activity in patients with community-acquired pneumonia (42). Compared to MP-treated patients that survived, MP-treated patients who died before 14 days presented with elevated oxidized glutathione at D5 when compared to survivors, indicating that this antioxidant system is key to prevent mortality even after MP treatment. In accordance, previous findings demonstrate that differences in glutathione metabolism are associated with fatal outcomes of COVID-19 patients (17).

Metabolites linked to tryptophan metabolism, FA metabolism, omega-6 metabolism, and TCA cycle were significantly altered, which is consistent with recent findings (12, 25, 40). Despite the MP intervention in the COVID-19 cohort, there were some fatalities. Comparison of the plasma metabolome perturbations between the placebo (survival) versus MP (death) cases suggested undesirable and potentially harmful effects that differed from person to person. These fatal outcomes were linked to changes in the vitamin D3 (cholecalciferol) metabolism, fatty acid activation, and glycerophospholipid metabolism pathways following the MP intervention. Caffeine, omega-6 FA metabolism, parathion degradation, and the generation of putative anti-inflammatory metabolites from EPA pathways were also observed. Vitamin D can help fight acute respiratory infections like COVID-19 and reduce their complications (43, 44). Our findings support the inverse relationship between corticosteroid use and serum vitamin D levels (45), whereby the MP may have caused significant changes in vitamin D metabolism, resulting in low 25-hydroxyvitamin D levels and an unfavorable outcome in some of the MP-treated patients. Our observation also suggests that modulating dysregulated Vitamin D metabolism is beneficial through controlling cytokine production and mitigating COVID-19 severity (25).

There were minimal changes between the D1 and D5 levels of butanoic acid, arginine, HTP, and serotonin in the surviving MP-treated patients. In contrast, butanoic acid and serotonin levels in placebo fatalities were higher at both time points than in the MP survivors, while arginine levels were lower and HTP levels alternated. Overall, there were substantial variations in these metabolites between the two treatment groups' outcomes. In our investigation, MP therapy improved patient survival by significantly suppressing (butanoic acid, HTP, and serotonin) and/or promoting (arginine), the expression of certain metabolites and/or their metabolism pathways. The considerable decrease of HTP and serotonin intensity levels, most likely due to tryptophan metabolism, highlights the complicated impact COVID-19 has on the immune system via kynurenine and nicotinamide pathways (12, 25, 46). The MP treatment appeared to protect against arginine depletion via arginine and proline metabolism, subsequently maintaining immune homeostasis (47), thereby promoting a favorable clinical outcome.

Integrating different orthogonal data sets in our study revealed the interconnection of different metabolite clusters with hematological, biochemical, and immunological components. Some of these metabolite clusters were unique for the different follow-up time points, whereas others were common in all. These indicate a crossroads of the different metabolic, physiological, and immunological processes involved in response to MP intervention in COVID-19. The integration of data on severity, fatality, and recovery from COVID-19 suggests that metabolic responses are an integrative communication hub of physiological processes in response to the SARS-CoV-2 infection (17). Although we did not distinguish between survivors and fatalities by D14 of follow-up, we believe that the significant metabolite clusters on D1, D5, D7, and D14 were the distinctive hallmarks of disease progression and therapeutic intervention. Metabolite clusters arising from metabolism pathways such as sialic acid and fructose/mannose, carnitine shuttle and phytanic acid peroxisomal oxidation, and arachidonate-derived prostaglandins and the TCA cycle pathways, for example, are positively linked with MP group at baseline, implying that they were suitable for corticosteroid function.

### Limitations

Our study has limitations. The first is the longitudinal loss in patient follow-up from either hospital discharge or in-hospital death. This temporal reduction in the sample size was coupled with gaps in laboratory data for some patients during follow-up. Second, some data, such as risky lifestyle behavior and previous use of other medications, were self-reported and may have been underestimated. In addition, comorbidities and previous use of medications, residual confounding from the patient’s diet and host genetics (48), may have affected the overall metabolomic profile. Finally, the high daily dose of corticosteroid administered can induce metabolic responses such as metabolic acidosis and hyperglycemia, which influence the activities of metabolic pathways.

### Conclusion

Corticosteroids have been pivotal in mitigating clinical COVID-19 symptoms. Similarly, metabolomics has proven to be valuable in unraveling and comprehending COVID-19. Overall, our findings suggest that treating SARS-CoV-2 infection with MP induced longitudinal changes in eight metabolic pathways, affecting various metabolites. Levels of butanoic acid, arginine, hydroxy-tryptophan, and serotonin may predict the 14 days outcome when treating COVID-19 with methylprednisolone. These results also support the need to consider other orthogonal data to evaluate the impact of using corticosteroids in managing COVID-19 patients. Future robust studies with longer follow-up times, different doses, and more events are warranted in positioning methylprednisolone/corticoids in treating coronavirus infections.

## Data Availability

Metabolomics data have been deposited in the Metabolomics Workbench database with the identifier ST002845.
